# A Laterally Vibrating Lithium Niobate MEMS Resonator Array Operating at 500 °C in Air

**DOI:** 10.3390/s21010149

**Published:** 2020-12-29

**Authors:** Savannah R. Eisner, Cailin A. Chapin, Ruochen Lu, Yansong Yang, Songbin Gong, Debbie G. Senesky

**Affiliations:** 1 Department of Electrical Engineering, Stanford University, 350 Serra Mall, Stanford, CA 94305, USA; 2 Department of Aeronautics and Astronautics, Stanford University, 496 Lomita Mall, Stanford, CA 94305, USA; cchapin3@stanford.edu (C.A.C.); dsenesky@stanford.edu (D.G.S.); 3 Department of Electrical and Computer Engineering, University of Illinois at Urbana-Champaign, 306 N Wright St, Urbana, IL 61801, USA; rlu10@illinois.edu (R.L.); yyang165@illinois.edu (Y.Y.); songbin@illinois.edu (S.G.)

**Keywords:** lithium niobate, RF MEMS, piezoelectric resonators, high-temperature, SH0 mode

## Abstract

This paper reports the high-temperature characteristics of a laterally vibrating piezoelectric lithium niobate (LiNbO_3_; LN) MEMS resonator array up to 500 °C in air. After a high-temperature burn-in treatment, device quality factor (*Q*) was enhanced to 508 and the resonance shifted to a lower frequency and remained stable up to 500 °C. During subsequent in situ high-temperature testing, the resonant frequencies of two coupled shear horizontal (SH0) modes in the array were 87.36 MHz and 87.21 MHz at 25 °C and 84.56 MHz and 84.39 MHz at 500 °C, correspondingly, representing a −3% shift in frequency over the temperature range. Upon cooling to room temperature, the resonant frequency returned to 87.36 MHz, demonstrating the recoverability of device performance. The first- and second-order temperature coefficient of frequency (TCF) were found to be −95.27 ppm/°C and 57.5 ppb/°C^2^ for resonant mode A, and −95.43 ppm/°C and 55.8 ppb/°C^2^ for resonant mode B, respectively. The temperature-dependent quality factor and electromechanical coupling coefficient (*k_t_*^2^) were extracted and are reported. Device *Q* decreased to 334 and total *k_t_*^2^ increased to 12.40% after high-temperature exposure. This work supports the use of piezoelectric LN as a material platform for harsh environment radio-frequency (RF) resonant sensors (e.g., temperature and infrared) incorporated with high coupling acoustic readout.

## 1. Introduction

Radio-frequency (RF) components capable of operating within environments extreme in temperature, pressure, corrosion, and radiation are desirable in a variety of industries, including aerospace, military, automotive, and energy harvesting. For instance, reliable devices that can combine passive RF/wireless signal processing with sensing modalities are particularly crucial to the length and scope of many proposed space missions, such as exploration of hostile planets like Venus (surface temperature 465 °C). Such devices could potentially eliminate the need for active electronics for in-sensor signal processing and seamlessly integrate the sensing and wireless readout in one battery-less package. To this end, piezoelectric RF microelectromechanical systems (MEMS) devices are particularly promising, as they offer compact and uncooled sensors monolithically integrated with acoustic readout devices that can survive on hot planets, as well as other Earth-based systems (e.g., oil, gas, and geothermal) [1,2,3,4].

Several piezoelectric devices fabricated with materials such as langasite (La_3_Ga_5_SiO_14_), aluminum nitride (AlN), and gallium nitride (GaN) have been examined for high-temperature RF applications [5,6,7,8,9,10,11,12]. Yet, the effective electromechanical coupling of the devices reported in these demonstrations is limited (*k_t_*^2^ < 1.4%), due to the inherently low piezoelectric coupling coefficients (*K^2^*) in the selected materials. The performance of piezoelectric lithium niobate (LiNbO_3_; LN) surface acoustic wave (SAW) devices was investigated at elevated temperatures as high as 600 °C in air, due to the attractive material properties of stoichiometric LN, such as a high Curie point of 1140 °C, chemical inertness, and the ability to maintain its favorable piezoelectric properties up to 750 °C [13,14,15,16,17,18]. However, the performance of LN SAW devices is still limited by only moderate coupling (*k_t_*^2^ ≈ 1.5%) and energy leakage into the substrate [19]. The low coupling and high loss in the aforementioned platforms fundamentally limit the bandwidth, signal-to-noise ratio, and the multiplexing capabilities needed for effective and robust passive wireless readout of sensor data at elevated temperatures.

Therefore, to address the need for low-loss, high coupling, and harsh environment capable sensors with integrated wireless readout, we examine the use of LN laterally vibrating resonators (LVRs). A distinct advantage of the LN LVR is its high electromechanical coupling coefficient (*k_t_*^2^ up to 30%) [20]. High *k_t_*^2^ values, along with comparable quality factors to other materials, can result in a higher figure of merit (*k_t_*^2^ × *Q*), lower insertion loss, and wider bandwidths in LN acoustic devices. In this paper, we present the high-temperature characteristics of an array of LN LVRs up to 500 °C in air. The laterally vibrating LN MEMS device exhibits recoverability after 500 °C exposure, a high temperature coefficient of frequency (TCF), and high *k_t_*^2^, demonstrating the potential of this material platform for low-loss and ultra-sensitive extreme environment remote RF sensing.

## 2. Device Design and Experimental Setup

**Figure 1:**
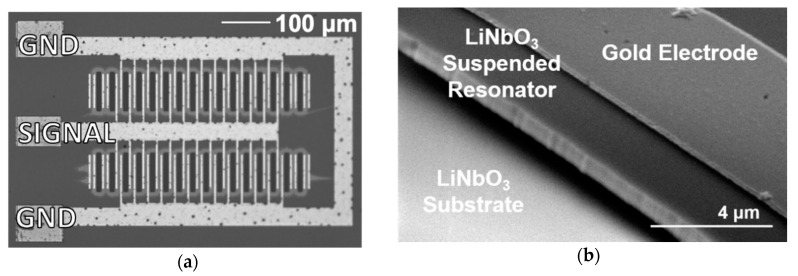
The microfabricated LN MEMS LVR array. Top-view optical image (**a**) and tilted SEM image of an individual resonator within the array (**b**).

Optical and scanning electron microscopy (SEM) images of the microfabricated LN LVR array are shown in Figure 1. The one-port array consists of 26 identical LVRs in parallel. Each individual resonator has two 100-nm-thick gold interdigitated electrodes, acting as signal and ground, on 700-nm-thick suspended stoichiometric LN. The resonator array was fabricated on an X-cut LN epitaxial thin film on nonstoichiometric LN substrate at −10° to the *Y*-axis, to excite the shear horizontal mode (SH0). The total device dimensions are 728 μm by 440 μm. Further fabrication and design details can be found in prior work [21,22].

**Figure 2:**
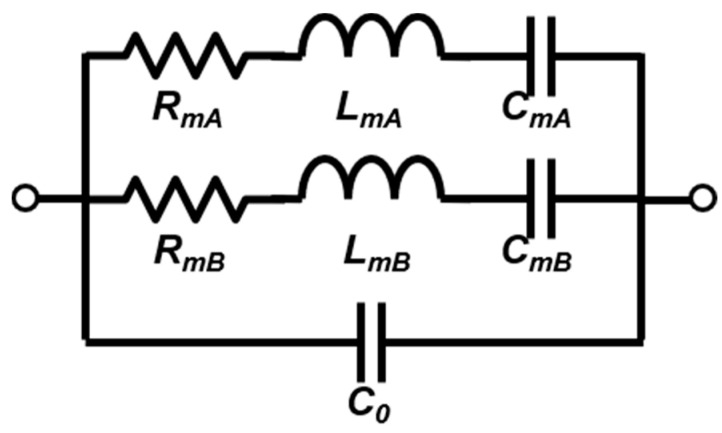
Modified Butterworth-Van Dyke (MBVD) model for the array at 25 °C, after the 500 °C burn-in.

The modified Butterworth-Van Dyke (MBVD) model obtained from the 25 °C post burn-in device admittance measurements is depicted in Figure 2. The MBVD model has two motional branches that represent two coupled SH0 modes very close to each other in resonance that were exhibited by the device after burn-in and during testing up to 500 °C [22,23]. The appearance of a second mode is attributed to a slight variation in mechanical boundary conditions, and thus resonant frequencies, among individual resonators in the array [22]. This variation could be due to particle mass loading on the day of testing, or non-uniform changes in the residual thin film stress due to temperature cycling. The extracted parameters of both modes, denoted as mode A (*R_mA_*, *L_mA_*, *C_mA_*) and mode B (*R_mB_*, *L_mB_*, *C_mB_*), are listed in Table 1.

**Figure 3:**
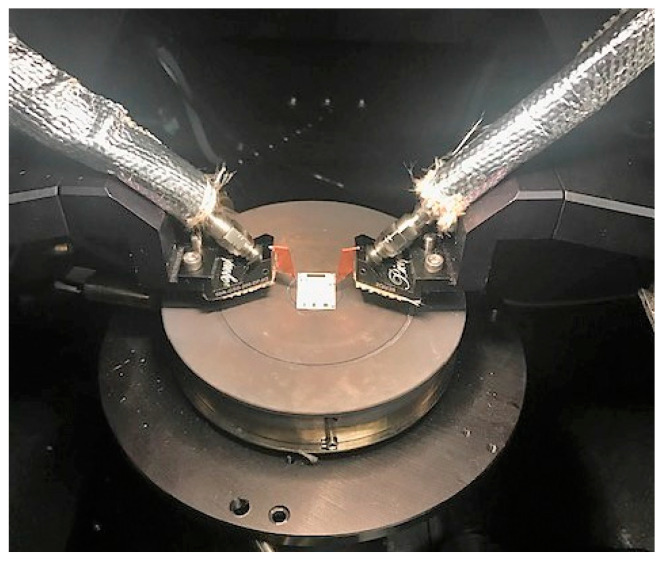
Photograph of the high-temperature measurement setup.

The experimental setup consisted of a network analyzer (Agilent E5063A) connected by 50 Ω impendence coaxial cable to a custom high-temperature ground-signal-ground (GSG) probe (Supplier: GGB Industries, Naples, FL, USA). As a further precaution against detrimental heating effects on the system, aluminized heat shielding was affixed to the bottom of the probe body and wrapped around the coaxial cable region closest to the probe. The probe was housed within an open-air high-temperature probe station (Supplier: Signatone Inc., Gilroy, CA, USA) equipped with a proportional-integral-derivative (PID) controlled heated chuck. The probe station setup was contained within a dark box and the door was shut during measurements. This prevented air flow throughout the room from causing local temperature variation on the sample. As per the heated chuck data sheet, the temperature accuracy and temperature uniformity were both ±1 °C. The temperature resolution of the PID controller was 1 °C. Figure 3 shows a photograph of the heat-shielded probes touching down on the ceramic calibration substrate on the heated chuck.

Using this setup, the resonator array was first subjected to a burn-in cycle up to 500 °C and back to room temperature in air. The purpose of the burn-in treatment was to stabilize any annealing effects (e.g., alloying due to diffusion of metal contacts) in the device. Initial and post-burn-in data was acquired. The device performance was then characterized in situ from 25 °C to 500 °C in 100 °C increments. To avoid thermal shock, the sample was gradually heated by a ramp not exceeding 10 °C/minute. To allow for sample temperature stabilization, the device was held at each temperature for twenty minutes before data were acquired. With this slow ramp and long soak time, the sample surface was assumed to have reached equilibrium with the heated chuck temperature by the time the measurements were taken.

After cooling back down from 500 °C to 25 °C, the device was measured again at room temperature. This room temperature measurement taken after the in situ high-temperature cycling is denoted “post in situ” throughout this paper.

## 3. Experimental Results and Discussion

**Figure 4:**
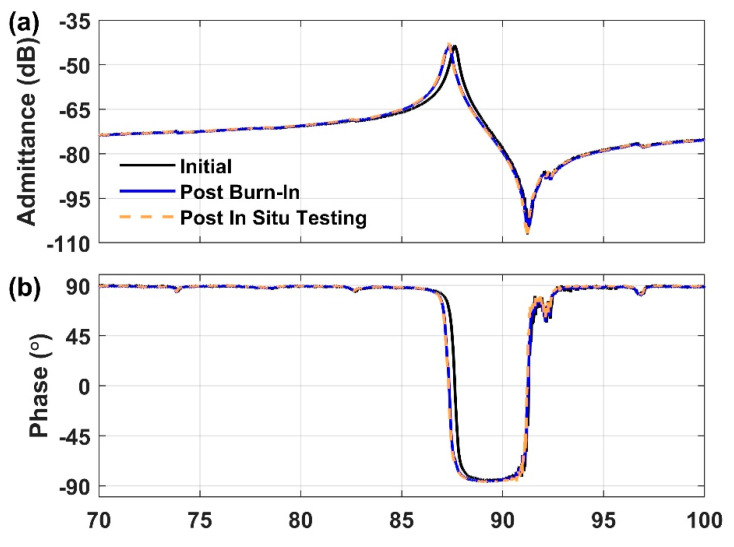

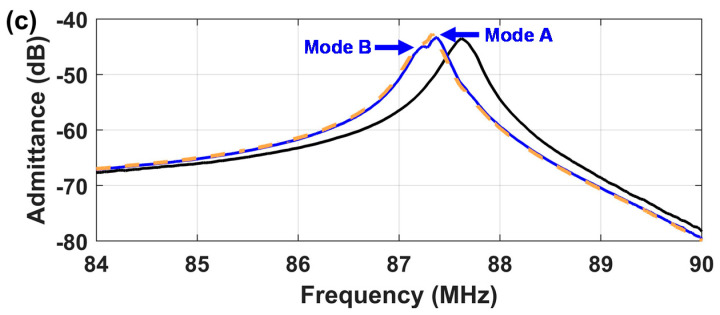
Measured amplitude (**a**) and phase (**b**) of the admittance of the LN MEMS LVR array at 25 °C initially, post burn-in, and post in situ testing up to 500 °C and back. Amplitude zoomed in around resonance and modes A and B in the post burn-in spectrum are labeled (**c**).

Figure 4 shows the measured admittance (Y_11_) of the LN MEMS LVR array initially, post burn-in, and post in situ testing. Extracted device parameters are listed in Table 2. During initial and post in situ testing, only one SH0 mode was measured and extracted, attributed to the mechanical boundary variations (see Device Design and Experimental Setup). Two distinct peaks, mode A and mode B, could be seen in the post burn-in spectrum at different frequencies (blue line in Figure 4c; modes labelled). After the burn-in temperature cycle, the resonance peak shifted down in frequency. The peak experienced a negligible shift after the in-situ testing up to 500 °C and back (Figure 4c; Table 2). This indicates that the burn-in stabilized the resonant frequency, likely due to an annealing effect on the gold electrodes. Yet, while the burn-in procedure enhanced the quality factor, *Q*, subsequent in situ high-temperature testing reduced *Q* below its initial value (Table 2). The *k_t_*^2^ of the array increased by a small amount from the initial to post in situ device testing (Table 2). The changes in *Q* and *k_t_*^2^ were likely caused by temperature-induced changes in the residual stress introduced during the fabrication process.

**Figure 5:**
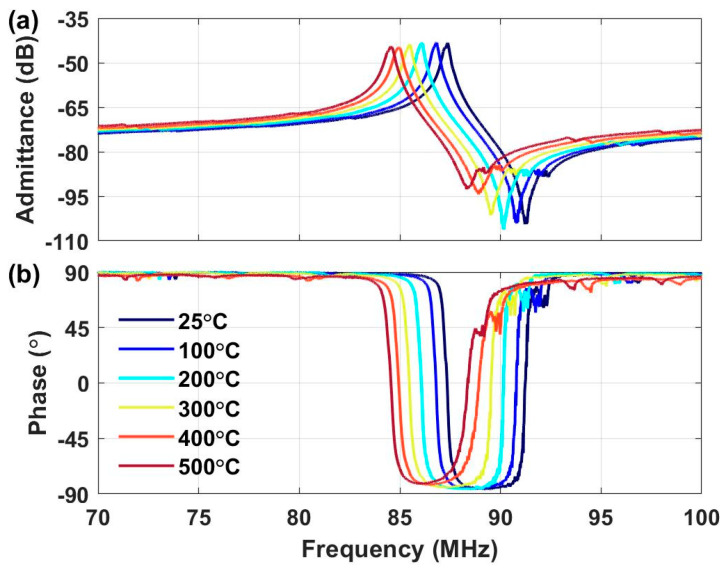
Measured amplitude (**a**) and phase (**b**) of the admittance of the LN MEMS LVR array from 25 °C to 500 °C.

The scattering parameter (S_11_) of the LN LVR array was measured in situ during temperature ramp up from 25 °C to 500 °Cand normalized to 50 Ω impedance. The admittance was determined from the scattering parameter and is plotted over the temperature and frequency range in Figure 5. Both the magnitude and phase of the admittance experience a downward shift in frequency from 25 °C to 500 °C, due to material softening. The stiffness constants of LN have negative temperature coefficients, indicating that the material softens with increasing temperature [24]. As the temperature is raised and the material softens, the elastic properties and the wave velocity are reduced, causing the resonant frequency to shift towards a lower value.

**Figure 6:**
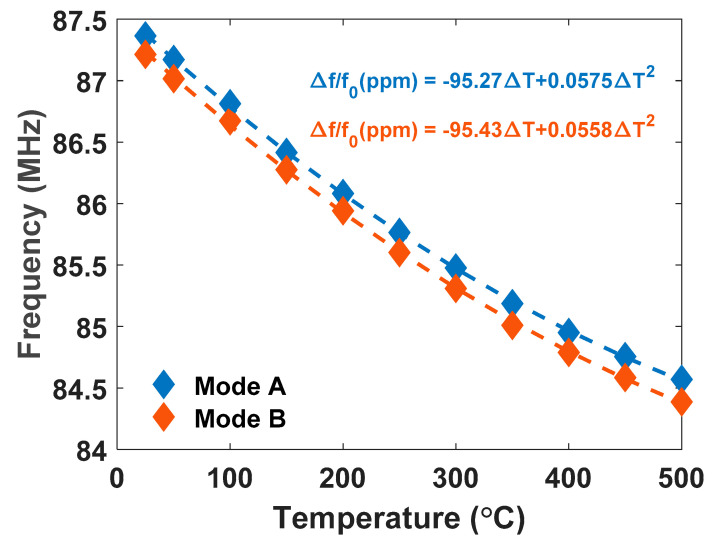
Measured temperature dependence of the shear horizontal (SH0) mode resonant frequencies up to 500 °C.

Figure 6 reports the temperature-dependent resonant frequency over the temperature range for both coupled SH0 modes A and B. The resonance of device mode A was 87.36 MHz at 25 °C and 84.56 MHz at 500 °C. The resonance of device mode B was 87.21 MHz at 25 °C and 84.39 MHz at 500 °C. This represents a −3.2% resonance shift for both modes. Upon cooling to room temperature, the resonant frequency returned to 87.30 MHz and only one mode was present, likely due to mechanical changes in the array after temperature cycling. The recoverability of resonance to within 0.1% of its post burn-in value after the high-temperature in situ exposure is promising (Table 2). Further temperature cycling studies are needed to determine whether the two shear horizonal modes permanently converged to one mode after the in situ testing.

Experimental frequency versus temperature results were best fit with quadratic equations and are displayed on Figure 6 as dashed lines. Using 25 °C as the reference temperature, the fractional frequency variation for mode A can be expressed as follows [9]:

(1) $$\frac{\Delta f}{f_{0}}=\;\frac{f\left(T\right)-f\left(25\right)}{f\left(25\right)}=\;-95.27\;\times \;10^{-6}\left(T-25\right)+\;57.5\;\times \;10^{-9}{\left(T-25\right)}^{2}\;$$

where *T* is temperature in degrees Celsius. The frequency variation can be similarly expressed for mode B and is shown in Figure 6. The R^2^ value of the fit was 0.9998. For mode A, the extracted first- and second-order temperature coefficient of frequency (TCF) were −95.27 ppm/°C and 57.5 ppb/°C^2^, respectively. The quadratic fit indicates an expected turnover temperature of 854 °C in air. For mode B, the first- and second-order TCF were −95.43 ppm/°C and 55.8 ppb/°C^2^, respectively. First-order TCF values were near the reported range for LN LVRs of −50 ppm/°C to −90 ppm/°C [24,25]. The TCF is a composite parameter that describes the change in frequency with temperature and captures the effects of material softening, linear thermal expansion, and the temperature coefficient of permittivity, among other mechanisms [26]. The large TCF implies high-resolution temperature sensing capabilities in LN LVRs compared to AlN and GaN piezoelectric thin film resonators, which exhibit uncompensated TCFs from −24 ppm/°C to −30 ppm/°C [1,8]. The second-order TCF largely depends on the temperature derivatives of higher order elastic coefficients, which are not currently known for lithium niobate. Reported values were not found in existing literature for comparison to the second-order TCF values extracted in this work.

**Figure 7:**
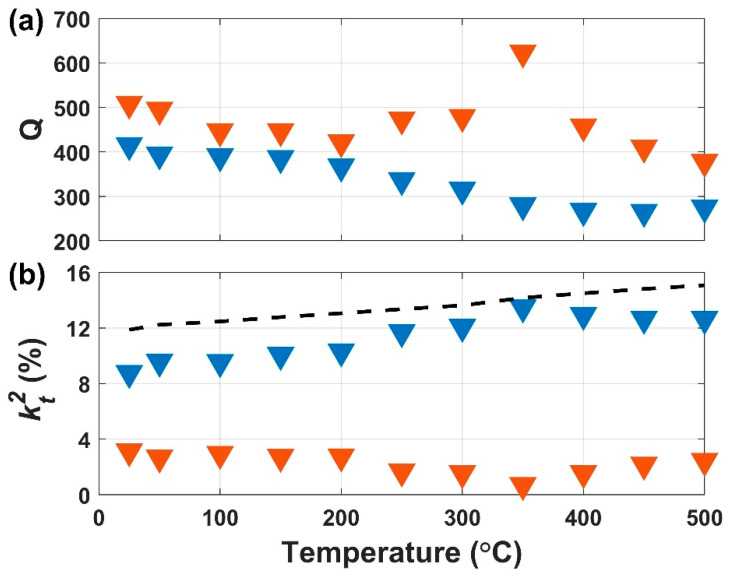
Extracted *Q* (**a**) and *k_t_*^2^ (%) (**b**) as a function of temperature. Mode A and B are represented by blue and orange triangles, respectively. The total *k_t_*^2^ (sum of both modes) is represented by the dashed black line.

The extracted *Q* and *k_t_*^2^ of device modes A and B at each temperature are shown in Figure 7a,b, respectively. The *Q* of mode A exhibits a steady 33% decrease with temperature, from 415 at 25 °C to 274 at 500 °C. The *Q* of mode B fluctuates with temperature, but decreases by 25% from 508 to 377 over the temperature range. The temperature dependence of *Q* is set by the energy loss mechanisms that are dominant within a micromechanical resonator’s regime of operation (e.g., temperature, pressure, and ambient environment). The total device quality factor, *Q_total_*, is related to each individual energy loss mechanism present within the micromechanical structure

(2) $$\frac{1}{Q_{total}}=\;\underset{i}{\sum }\frac{1}{Q_{i}}$$

where *Q_i_* is the quality factor associated with a particular mechanism [26]. This indicates that the mechanism with the lowest individual *Q_i_* will fundamentally limit *Q_total_*. Well-known loss mechanisms include air damping, thermoelastic dissipation (TED), phonon–phonon dissipation (Akhiezer effect), anchor loss, and electrical losses. In this work, we report the overall temperature dependency of *Q*. Identifying the dominant *Q*-limiting loss mechanisms was not the focus of the work and requires further investigation in a future study. The fluctuations in Q were attributed to mechanical boundary changes in individual resonators within the array, during thermal exposure.

The *k_t_*^2^ of boths modes changed during the temperature rise (Figure 7b). This fluctuation of coupling in individual modes could be explained by the slight differences in TCF between the two modes. However, the sum of *k_t_*^2^ for both modes steadily increased, rising from 11.9% at 25 °C to 15.1% at 500 °C. The rise in total electromechanical couplingis due to the positive temperature coefficients of the piezoelectric coupling constants of LN [24].

## 4. Conclusions

This article presents the experimental results of high-temperature (500 °C) operation of a laterally vibrating (shear horizontal mode) lithium niobate MEMS resonator array. The lithium niobate laterally vibrating resonator device displayed recoverability of resonant frequency after 500 °C air exposure. The large first-order frequency variation with temperature of (−95 ppm/°C TCF) supports the use of uncompensated lithium niobate laterally vibrating resonators as high-sensitivity extreme environment sensors (e.g., infrared sensors for space exploration and other high-temperature applications). The high and stable total electromechanical coupling coefficient, *k_t_*^2^, was 15% at 500 °C and 12.4% upon return to room temperature, demonstrating the potential for low insertion loss, wide bandwidth lithium niobate laterally vibrating acoustic delay lines to be integrated with resonant sensors for passive wireless readout of sensor data at high-temperature.

## Figures and Tables

**Table 1:** Extracted values for the MDVD model shown in Figure 2.

Parameter	Mode A	Mode B
*R_m_* (Ω)	166	383
*C_m_* (fF)	26.37	9.39
*L_m_* (μH)	126	355
*C*_0_ (fF)	371	

**Table 2:** Extracted key parameter values initially, post burn-in and post in situ testing as shown in Figure 4.

Parameter	Initial	Post Burn-In (Mode A/B)	Post In Situ Testing
*f_s_* (MHz)	87.62	87.36/87.21	87.30
*Q*	348	415/508	334
*k_t_*^2^*(*%*)*	11.39	8.75/3.12	12.40
